# Blocking of PDL-1 Interaction Enhances Primary and Secondary CD8 T Cell Response to Herpes Simplex Virus-1 Infection

**DOI:** 10.1371/journal.pone.0039757

**Published:** 2012-07-12

**Authors:** Rudragouda Channappanavar, Brandon S. Twardy, Susmit Suvas

**Affiliations:** Department of Biological Sciences, Oakland University, Rochester, Michigan, United States of America; Dartmouth Medical School, United States of America

## Abstract

The blocking of programmed death ligand-1 (PDL-1) has been shown to enhance virus-specific CD8 T cell function during chronic viral infections. Though, how PDL-1 blocking at the time of priming affects the quality of CD8 T cell response to acute infections is not well understood and remains controversial. This report demonstrates that the magnitude of the primary and secondary CD8 T cell responses to herpes simplex virus-1 (HSV-1) infection is subject to control by PDL-1. Our results showed that after footpad HSV-1 infection, PD-1 expression increases on immunodominant SSIEFARL peptide specific CD8 T cells. Additionally, post-infection, the level of PDL-1 expression also increases on CD11c+ dendritic cells. Intraperitoneal administration of anti-PDL-1 monoclonal antibody given one day prior to and three days after cutaneous HSV-1 infection, resulted in a marked increase in effector and memory CD8 T cell response to SSIEFARL peptide. This was shown by measuring the quantity and quality of SSIEFARL-specific CD8 T cells by making use of *ex-vivo* assays that determine antigen specific CD8 T cell function, such as intracellular cytokine assay, degranulation assay to measure cytotoxicity and viral clearance. Our results are discussed in terms of the beneficial effects of blocking PDL-1 interactions, while giving prophylactic vaccines, to generate a more effective CD8 T cell response to viral infection.

## Introduction

An optimum CD8 T cell response to viral infection is dependent upon T-cell receptor (TCR) stimulation along with costimulatory signals [1]. Dysregulation in positive and negative co-stimulus affects the outcome of T cell activation and proliferation [1]. Recently, it has been shown that programmed death-1 (PD-1), an inhibitory receptor, is highly expressed on dysfunctional virus specific CD8 T cells during several chronic viral infections [2], [3], [4], [5]. PD-1 interacts with its ligand PDL-1 and PDL-2 [6]. Blocking of PD-1: PDL-1 interaction enhances the quality of virus specific CD8 T cells during chronic lymphocytic choriomeningitis virus (LCMV) and simian immunodeficiency virus (SIV) infections [2], [7]. In addition, *in vitro* blocking of PDL-1 interaction with PD-1 enhances the effector function of HIV and HCV specific CD8 T cells that were isolated from HIV and HCV infected individuals [4], [5], [8]. These studies clearly demonstrate that under chronic viral infection conditions, the PDL-1 blockade markedly enhances the proliferation, cytokine secretion and cytotoxic potential of viral antigen specific CD8 T cells.

Role of PD-1: PDL-1 interaction in regulating CD8 T cell function during acute infections is not clear and controversial. In a primary *Listeria monocytogenes* bacterial infection model, PDL-1 blocking, while administering anti-PDL-1 monoclonal antibody, impedes proliferation and effector function of antigen specific CD8 T cells resulting into the delayed bacterial clearance [9], [10]. On the other hand, blocking PDL-1 on Respiratory syncytial virus (RSV)-infected bronchial epithelial cells in *ex-vivo* cultures with CD8 T cells enhances IFN-γ, IL-2 and granzyme B production by antigen specific CD8 T cells [11]. Similarly, during acute hepatitis C virus (HCV) infection, PD-1 is up regulated on HCV-specific CD8 T cells and *ex-vivo* blocking of the PD-1: PDL-1 interaction improves the proliferation of virus specific CD8 T cells [12]. In contrast, during acute Friend Retrovirus infection, PD-1 expressing CD8 T cells were highly cytotoxic and blocking PDL-1 interactions did not enhance the effector function of CD8 T cell [13]. Therefore, the precise role of the PD-1: PDL1 interaction in regulating the generation of good-quality virus specific effector and memory CD8 T cell pool during acute viral infection remains to be fully defined.

Cutaneous infection with herpes simplex virus-1 (HSV-1) is an interesting localized infection model to study the HSV-1 specific CD8 T cell response [14]. In this report, we demonstrate that after footpad HSV-1 infection, PD-1 and PDL-1 expression increases on immunodominant SSIEFARL (gB_498–505_) peptide specific CD8 T cells and dendritic cells, respectively. Our results show that blocking the PD-1: PDL-1 interaction at the time of priming, while administering anti-PDL-1 antibody, markedly increases the absolute numbers of gB_498–505_ tetramer specific CD8 T cells. Moreover, blocking the PDL-1 interaction at the time of priming improves the cytotoxic potential and cytokine secreting ability of the virus specific effector and memory CD8 T cell pool. Taken together, our results determined that the magnitude of the primary and secondary CD8 T cell responses to immunodominant SSIEFARL peptide, after cutaneous HSV-1 infection, is subject to control by PDL-1 interaction with its ligand PD-1.

## Results

### PD-1 Expression on SSIEFARL Specific CD8 T Cells during Expansion and Contraction Phase of Primary CD8 T Cell Response to HSV-1 Infection

**Figure 1:**
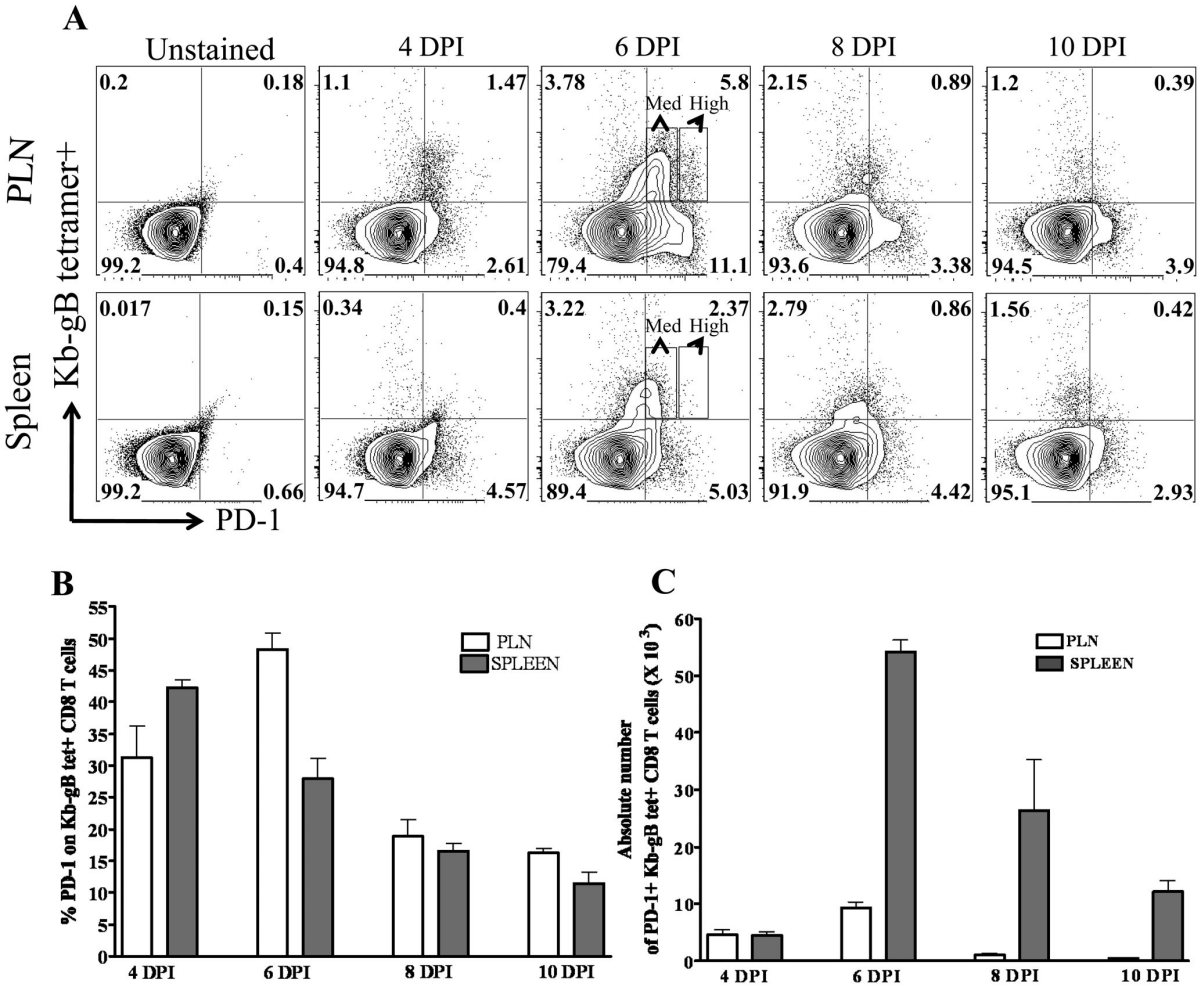
Kinetics of PD-1 expression on gB_498–505_ (SSIEFARL) peptide specific CD8 T cells in PLN and spleen tissue of HSV-1 infected mice. C57BL/6 mice were infected with 1×10^6^ p.f.u of HSV-1 (KOS) in the hind footpad. Mice were euthanized on day 4, 6, 8 and 10 post-infection. PD-1 expression on gB_498–505_ tetramer+ CD8 T cells was determined in the PLN and spleen by flow cytometery at each time-point. A, The representative FACS plots gated on CD8 T cell demonstrate the frequency of tetramer+ PD-1+ cells in PLN and spleen tissue at different time-points post-infection. PD-1^high^ and PD-1^medium^ expression on SSIEFARL specific CD8 T cells are shown at day 6 post-infection. B and C, The bar graphs represent the average percentage and absolute numbers of SSIEFARL tetramer+ CD8 T cells expressing PD-1 in PLN and spleen tissue of HSV-infected mice on different days post-infection. Data shown is derived from four mice at each time-point post-infection.

PD-1 is expressed within 24–72 h of antigenic stimulation of T cells [6]. We looked at the kinetics of PD-1 expression on immunodominant SSIEFARL (gB_498–505_) peptide specific CD8 T cells in the PLN and spleen tissue of C57BL/6 mice after footpad HSV-1 infection. As is shown in Figure 1A and B, on day six post-infection, in PLN an average of about 50% of tetramer positive CD8 T cells were expressing PD-1, whereas in the spleen more than 25% of tetramer positive CD8 T cells were PD-1 positive. The maximum numbers of PD-1 expressing tetramer positive CD8 T cells were observed on day six post-infection in PLN and spleen tissue of HSV-1 infected mice (Figure 1C). Additionally, as shown in Figure 1A, on day 6 post-infection, a differential level of PD-1 expression was noticed on SSIEFARL specific CD8 T cells largely in the popliteal lymph node. The majority of tetramer positive CD8 T cells were PD-1^medium^ whereas a small proportion of SSIEFARL specific CD8 T cells were expressing high levels of PD-1 (PD-1^high^). Similar trends of PD-1^high^ and PD-1^medium^ expression were also observed on non-SSIEFARL but possibly HSV-1 specific CD8 T cells in the PLN and spleen. By day 10 post-infection, PD-1 was down regulated in an average of more than 80% of tetramer positive CD8 T cells. Taken together, our results show that PD-1 expression is upregulated on SSIEFARL specific CD8 T cells during expansion, but gets down regulated during the contraction phase of the primary CD8 T cell response to HSV-1 infection.

### Cytotoxic Potential and Pro-inflammatory Cytokine Secreting Ability of PD-1^high^ and PD-1^medium^ CD8 T Cells after HSV-1 Infection

**Figure 2:**
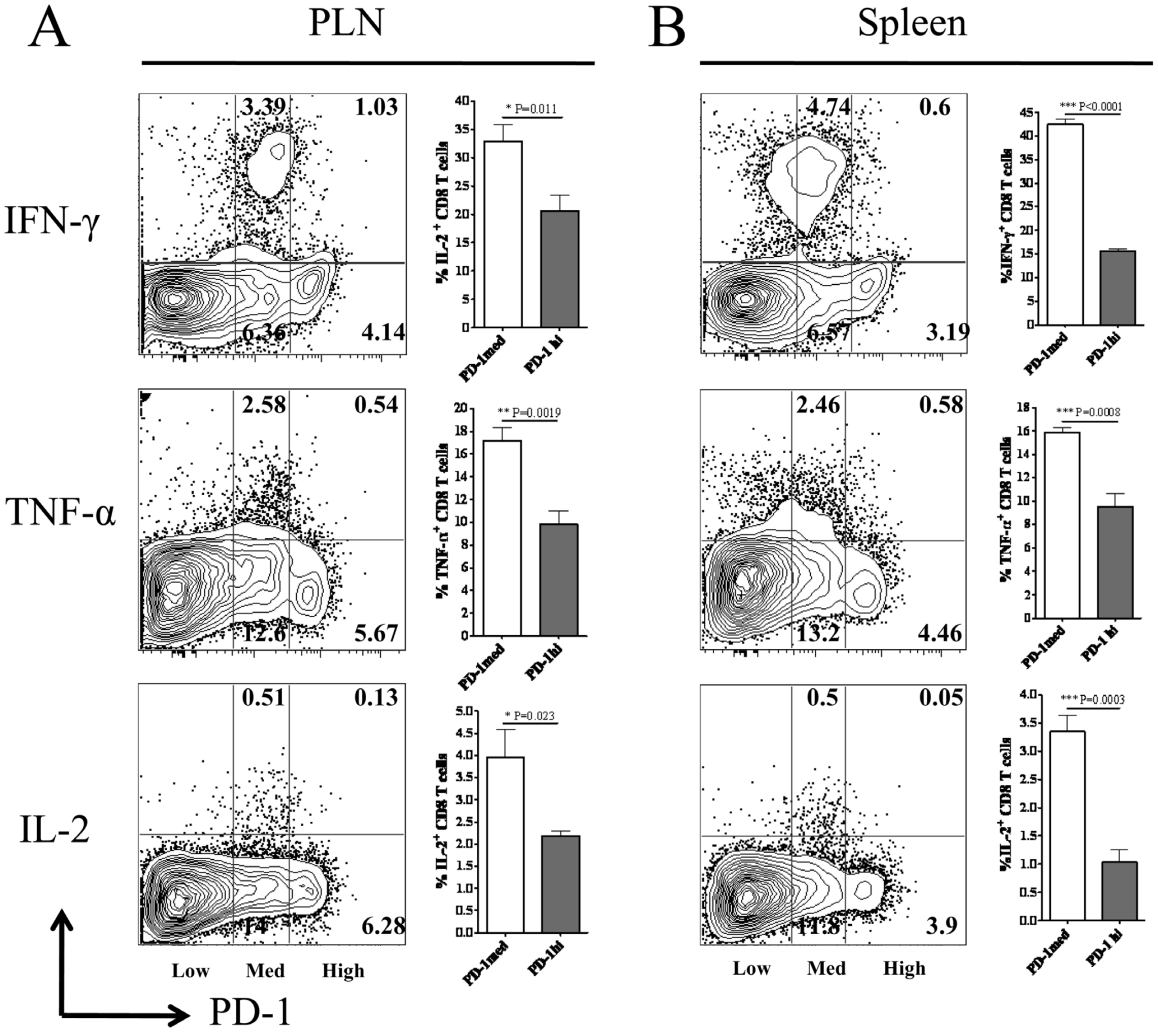
Cytokine producing ability of PD-1^high^ and PD-1^medium^ CD8 T cells during lytic phase of HSV-1 infection. Mice infected with HSV-1 were euthanized on day 6 post-infection. PLN and spleen tissue obtained were homogenized to obtain singe cell suspension. CD8 T cells were stimulated with immunodominant SSIEFARL peptide and pro-inflammatory cytokine producing PD-1^high^ and PD-1^medium^ CD8 T cells were determined by flow cytometery as described in the methods. A and B, Representative FACS plots gated on CD8 T cell and bar diagrams denote the proportion of IFN-γ, TNF-α and IL-2 producing PD-1^medium^ and PD-1^high^ CD8 T cells in PLN and spleen on day 6 post-infection, respectively. Data shown is representative of two similar experiments with five mice in each group/experiment. Statistical significance was determined with unpaired student’s t test where *p<0.05, **p<0.01 and ***p<0.0001 were considered significant.

**Figure 3:**
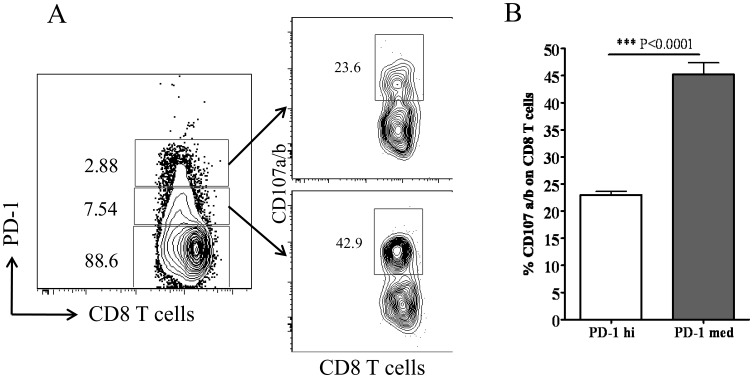
Granzyme B secreting potential of PD-1^high^ and PD-1^medium^ CD8 T cells in spleen during lytic phase of HSV-1 infection. *Ex-vivo* cytotoxicity of splenic PD-1^high^ and PD-1^medium^ CD8 T cells on day 6 post-infection was compared after stimulating with SSIEFARL peptide as described in the methods. A, The representative FACS plot denotes the ratio of PD-1^high^ (2.88), PD-1^medium^ (7.54) and PD-1^negative^ (88.6) splenic CD8 T cells on day 6 post-infection. The frequencies of cell surface expressed CD107a/b by PD-1^high^ and PD-1^medium^ CD8 T cells are shown in second column. B, The bar diagram shows the mean ± S.D. of the frequency of cell surface expressing CD107a/b PD-1^high^ and PD-1^medium^ CD8 T cells from five mice. Statistical significance was determined with unpaired student’s t test where ***p<0.0001 were considered statistically significant.

PD-1 signaling can down-regulate the cytotoxic potential and pro-inflammatory cytokine production by virus specific effector CD8 T cells [15]. Our results determined differential levels of PD-1 expression on HSV-1 specific CD8 T cells at the peak of the immune response to subcutaneous HSV-1 infection. Therefore, we next ascertained the cytotoxic potential and the ability of PD-1^high^ and PD-1^medium^ CD8 T cells to produce pro-inflammatory cytokines like IL-2, IFN-γ and TNF-α when stimulated *in vitro* with immunodominant SSIEFARL peptide on day six post-infection. As is shown in Figure 2, the frequency of PD-1^medium^ CD8 T cells secreting IL-2, IFN-γ and TNF-α cytokines in response to SSIEFARL stimulation was significantly higher when compared with PD-1^high^ CD8 T cells in PLN as well as in the spleen tissue of the infected mice. In addition, the proportion of PD-1 expressing (irrespective of the level of PD-1 expression) CD8 T cells producing pro-inflammatory cytokines was in the order of IFN-γ>TNF-α>IL-2 when measured in the above mentioned tissues. Similarly, as determined by a granzyme B degranulation assay, after *in vitro* stimulation with SSIEFARL peptide, the proportion of cell surface expressed CD107a/b molecules by PD-1^high^ was significantly less than PD-1^medium^ CD8 T cells obtained from the spleen tissue (Figure 3). No significant difference in the ratio of CD107 expressing PD-1^high^ and PD-1^medium^ CD8 T cells was noted in PLN after SSIEFARL stimulation (data not shown). These results determined that PD-1^high^ CD8 T cells in PLN as well as in spleen were more exhaustive in terms of their ability to secrete pro-inflammatory cytokines when compared to respective PD-1^medium^ CD8 T cells, whereas, PD-1^high^ CD8 T cells were less cytotoxic than PD-1^medium^ CD8 T cells in the spleen.

### PDL-1 Expression on CD11c+ Dendritic Cells in PLN after Foot-pad HSV-1 Infection

**Figure 4:**
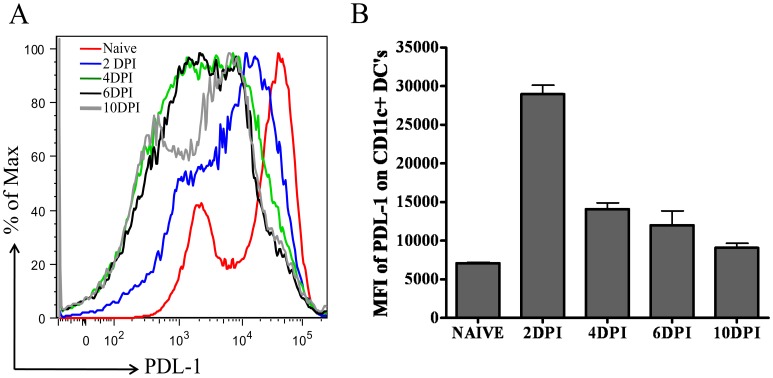
Kinetics of PDL-1 expression on CD11c+ dendritic cells derived from popliteal lymph node (PLN) of HSV-1 infected mice. A, A representative histogram overlay, obtained from gating on CD11c+ cells, denotes the expression of PDL-1 on CD11c+ dendritic cells in PLN at different time-points post-infection. Naïve represents level of PDL-1 expression on CD11c+ DCs in the PLN of uninfected mice. B, Bar graph shows mean ± S.D. of PDL-1 mean fluorescence intensity (MFI) on CD11c+ DCs in naive and infected mice at different time-points post-infection. Data shown is derived from four mice at each time-point.

PD-1 delivers inhibitory signals to T cells when it interacts with its ligand PDL-1 in the presence of TCR stimulation [6]. CD11c positive dendritic cells (DCs) are the key players in priming HSV-1 specific CD8 T cells in the PLN after footpad HSV-1 infection [16]. Therefore, we next determined the kinetics of PDL-1 expression on CD11c+ dendritic cells in the PLN of C57BL/6 mice by measuring the mean fluorescence intensity (MFI) of PDL-1 on gated CD11c+ cells at different time-points post-infection. As is shown in Figure 4, PDL-1 expression increases on CD11c+ cells in the PLN after footpad HSV-1 infection and the peak expression was noticed on day 2 post-infection followed by their down-regulation. By day 10 post-infection, cell surface expression of PDL-1 on DCs is nearer to its base-line expression in naïve mice. These results demonstrate that after footpad HSV-1 infection of C57BL/6 mice, PDL-1 is transiently upregulated on CD11c+ DCs in the draining lymph node of the infected mice.

### PDL-1 Blocking during T Cell Priming Increases the Proportion and Absolute Number of SSIEFARL Specific CD8 T Cells at the Peak of Acute Immune Response to HSV-1 Infection

**Figure 5:**
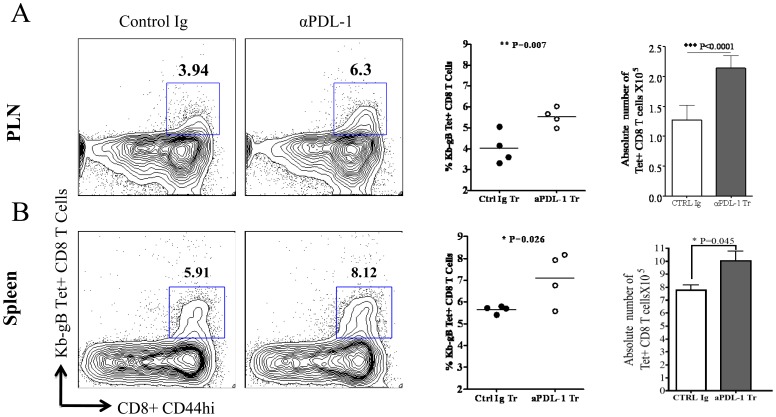
Anti-PDL-1 antibody treatment increases the frequency and absolute numbers of SSIEFARL specific CD8 T cell in PLN and spleen tissue of HSV-1 infected mice. Anti-PDL-1 or Isotype matched antibody was intra-peritoneally administered on -1 and 3 days post footpad HSV-1 infection. Both groups of mice were euthanized on day 6 post-infection and single cell suspensions from PLN and spleen tissue were cell surface stained with gB_498–505_ tetramer to detect immunodominant SSIEFARL peptide specific CD8 T cells. The representative FACS plots are gated on CD8 T cells and demonstrate the proportion of SSIEFARL specific CD8 T cells in isotype and anti-PDL-1 treated mice in PLN and spleen, respectively. The scatter plots represent average percentage and the bar diagram shows the average absolute numbers of SSIEFARL specific CD8 T cells in PLN and spleen tissue of isotype and anti-PDL-1 antibody treated groups of mice. Data shown is the representative of two independent experiments including 4mice/experiment. Statistical significance was determined by unpaired students t test with *P<0.05, **P<0.01 and ***P<0.0001 were considered statistically significant.

It has been shown that intra-peritoneal administration of anti-PDL-1 (10F.9G2) antibody result in the proliferation of virus specific memory CD8 T cells during chronic viral infection [2]. We observed a heightened level of PDL-1 on CD11c+ DCs in the draining lymph node of HSV-1 infected mice (Figure 4). Therefore, we ascertained whether blocking PDL-1 interactions during T cell priming enhances the frequency and numbers of immunodominant SSIEFARL specific CD8 T cells. The PDL-1 interaction with PD-1 was blocked by intra-peritoneal administration of anti-PDL-1 mAb (10F.9G2) given one day before and three days after subcutaneous HSV-1 infection. The isotype-matched antibody was administered in the control groups of infected mice. Mice from both groups were euthanized on day six post-infection. The proportion and the absolute numbers of SSIEFARL specific CD8 T cells in PLN and spleen tissue were determined by tetramer (gB_498–505_) staining for immunodominant SSIEFARL peptide. As is evident in Figure 5, anti-PDL-1 antibody treatment resulted in a statistically significant increase in the proportion of SSIEFARL specific CD8 T cells in PLN (p = 0.007) and spleen (p = 0.026) tissue versus the control infected group of mice. Similarly, the absolute numbers of SSIEFARL specific CD8 T cells were also greater in the PLN (p<0.0001) and spleen (p = 0.045) of mice treated with anti-PDL1 antibody than isotype treated infected group. Our results determined that PDL-1 blocking during T cell priming augments the frequency and absolute numbers of immunodominant SSIEFARL specific effector CD8 T cell in PLN and spleen tissue of HSV-1 infected mice at the peak of the primary CD8 T cell response to HSV-1 infection.

### Blocking of PDL-1 during T Cell Priming Increases the Frequency of IL-2, IFN-γ and TNF-α Producing Virus Specific Effector CD8 T Cells in PLN and Spleen Tissue of HSV-1 Infected Mice

**Figure 6:**
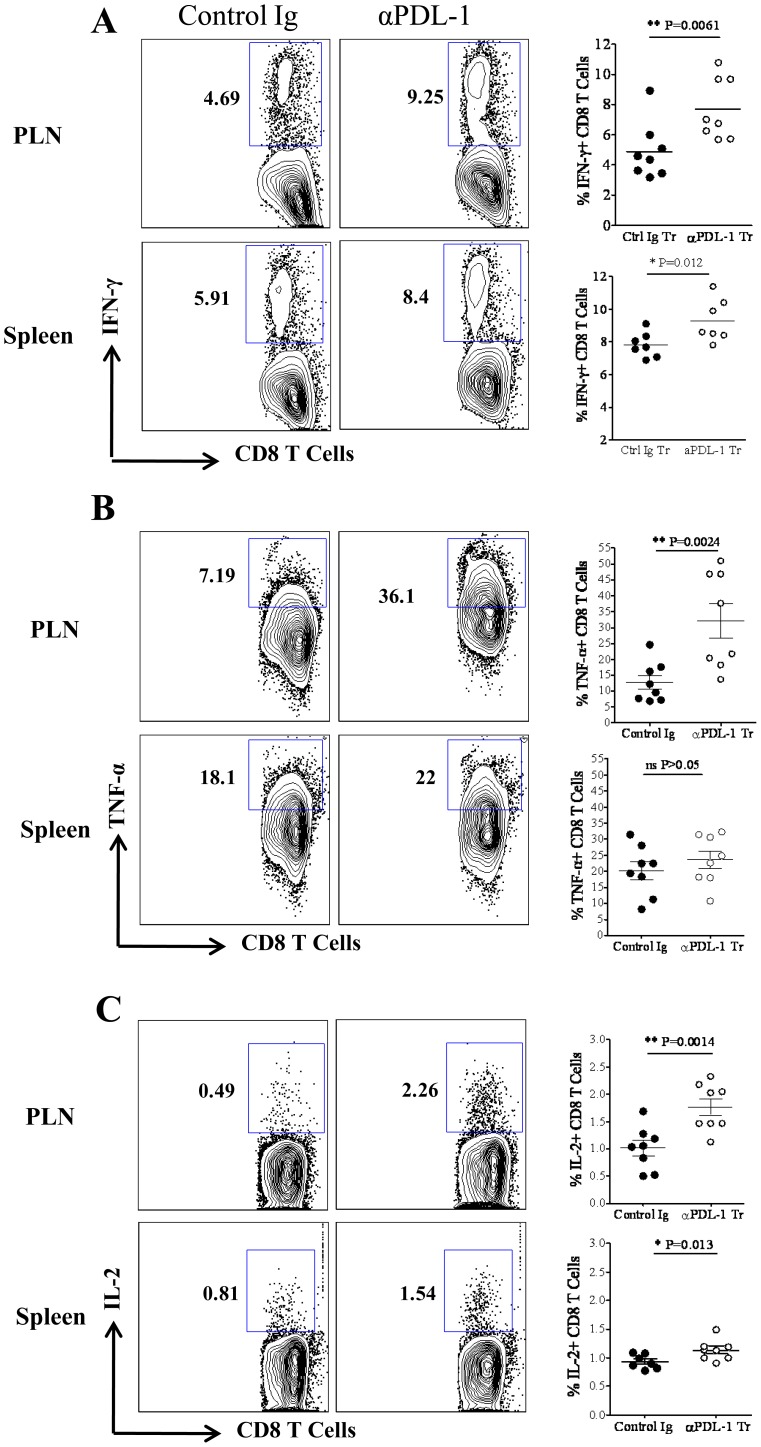
Anti-PDL-1 antibody treatment increases the proportion of IL-2, IFN-γ and TNF-α producing SSIEFARL specific effector CD8 T cells. Mice from anti-PDL-1 or isotype treated groups were euthanized on day 6 post infection. Single cell suspensions obtained from PLN and spleen tissue were stained intra-cellularly for pro-inflammatory cytokines after brief *in vitro* stimulation with SSIEFARL peptide (10 µg/ml). The representative FACS plots in A, B and C demonstrates the proportion of IFN-γ, TNF-α and IL-2 secreting CD8 T cells, respectively, in PLN and spleen tissue derived from isotype and anti-PDL-1 treated mouse. Scatter plots in panel A, B and C show mouse to mouse variation in the frequency of IFN-γ, TNF-α and IL-2 producing CD8 T cell in PLN and spleen tissue of isotype and anti-PDL-1 treated groups of mice. Data shown is derived from two similar experiments with four mice per group in each experiment. Statistical significance was determined by unpaired student’s t test where *p<0.05 and **p<0.01 are considered statistically significant.

Blocking of PDL-1 interaction with PD-1, while administering anti-PDL-1 antibody, is reported to enhance the effector function of exhausted memory CD8 T cells in chronic viral infections [2]. We thus determined whether the blocking PDL-1 interaction at the time of priming increases the proportion of cytokine secreting SSIEFARL specific CD8 T cells during the acute phase of immune response to HSV-1 infection. Anti-PDL-1 antibody was given one day before and three days post foot-pad HSV-1 infection. Control groups of infected mice received an isotype-matched antibody at the same time-points. Both groups of mice were euthanized at day six post-infection and single cell suspensions of PLN and spleen tissue were stimulated *in vitro* with immunodominant SSIERARL peptide to determine the frequency of cytokine secreting viral antigen specific CD8 T cells. Our results showed a statistically significant increase in the frequency of IFN-γ secreting SSIEFARL specific CD8 T cells in the PLN (p = 0.0061) and spleen (p = 0.012) tissue of the anti-PDL1 treated group of mice (Figure 6A). In addition, there was an average of more than a two fold increase in the proportion of TNF-α secreting virus specific effector CD8 T cells in the PLN of the anti-PDL-1 treated group of mice compared to the isotype treated infected group (Figure 6B). Similarly, more IL-2 secreting virus specific CD8 T cells were observed in the PLN and spleen tissue of the anti-PDL-1 treated group when compared with the isotype treated infected group (Figure 6C). Thus, our results determined a significant increase in the proportion of IL-2, IFN-γ and TNF-α secreting SSIEFARL specific effector CD8 T cells after blocking the PDL-1 interaction at the time of priming the CD8 T cell response to HSV-1 infection.

### Blocking of PDL-1 Enhances the Cytotoxic Potential of SSIEFARL Specific CD8 T Cells in Lymphoid Tissue

**Figure 7:**
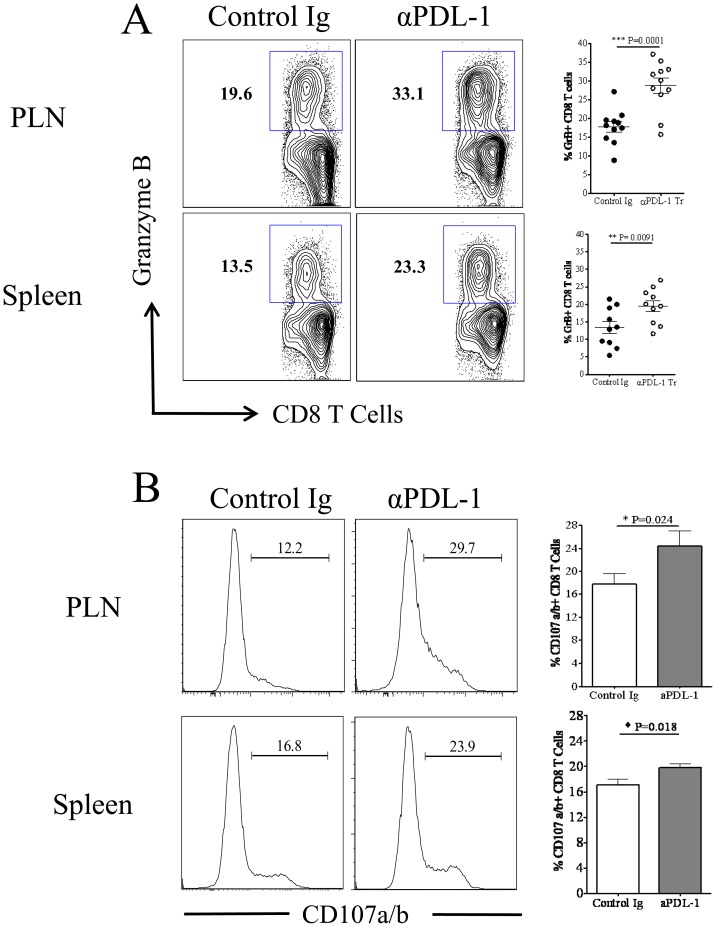
Anti-PDL-1 treatment increases the cytotoxic potential of HSV-1 specific effector CD8 T cell in the PLN and spleen tissue during lytic phase of HSV-1 infection. Mice from isotype and anti-PDL1 treated groups were euthanized on day 6 post-infection. Single cell suspensions obtained from PLN and spleen tissue were *ex-vivo* stimulated with SSIEFARL peptide in the presence of fluorochrome conjugated anti-CD107a/b antibodies followed by intra-cellular staining for granzyme B molecule. A, Representative FACS plots and scatter plots denotes the proportion of granzyme B expressing CD8 T cells in PLN and spleen tissue of control and anti-PDL1 treated groups on day 6 post-infection. B, Histogram FACS plots, derived from gated CD8 T cell, and the bar diagram demonstrate the frequency of CD8 T cells expressing membrane bound CD107a/b in PLN and spleen tissue of control and anti-PDL1 treated groups of mice. Data shown is derived from two similar experiments with four mice per group in each experiment. Statistical significance was determined by unpaired two-tailed student’s t test where *p<0.05 and **p<0.01 are considered statistically significant.

The cytotoxic potential of SSIEFARL specific CD8 T cells in the isotype and anti-PDL-1 treated groups of mice was compared by determining the proportion of intra-cellular granzyme B and cell surface CD107a/b expressing CD8 T cells at day 6 post-infection. CD107a/b is expressed onto the cell surface of CD8 T cells after the later exocytose granzyme B molecule. As is shown in Figure 7A, anti-PDL-1 antibody treatment significantly enhanced the frequency of granzyme B expressing CD8 T cells both in PLN and spleen tissue on day 6 post-infection. In addition, when measuring the ability of CD8 T cells to exocytose intracellular granzyme B molecules in an *ex-vivo* SSIEFARL stimulating assay, our results determined higher percentage of SSIEFARL specific CD8 T cells expressing cell surface CD107a/b molecule in the PLN and Spleen tissue of anti-PDL-1 treated group of mice than isotype treated infected group suggesting more exocytosis of granzyme B in response to blocking of PDL-1 interaction (Figure 7B). Thus, *in vivo* blocking of PDL-1 improves the cytotoxic potential of immunodominant SSIEFARL specific CD8 T cells during acute phase of CD8 T cell response to HSV-1.

### PDL-1 Blocking at the Time of CD8 T Cell Priming Decreases Viral Load in the Foot-pad Tissue

**Figure 8:**
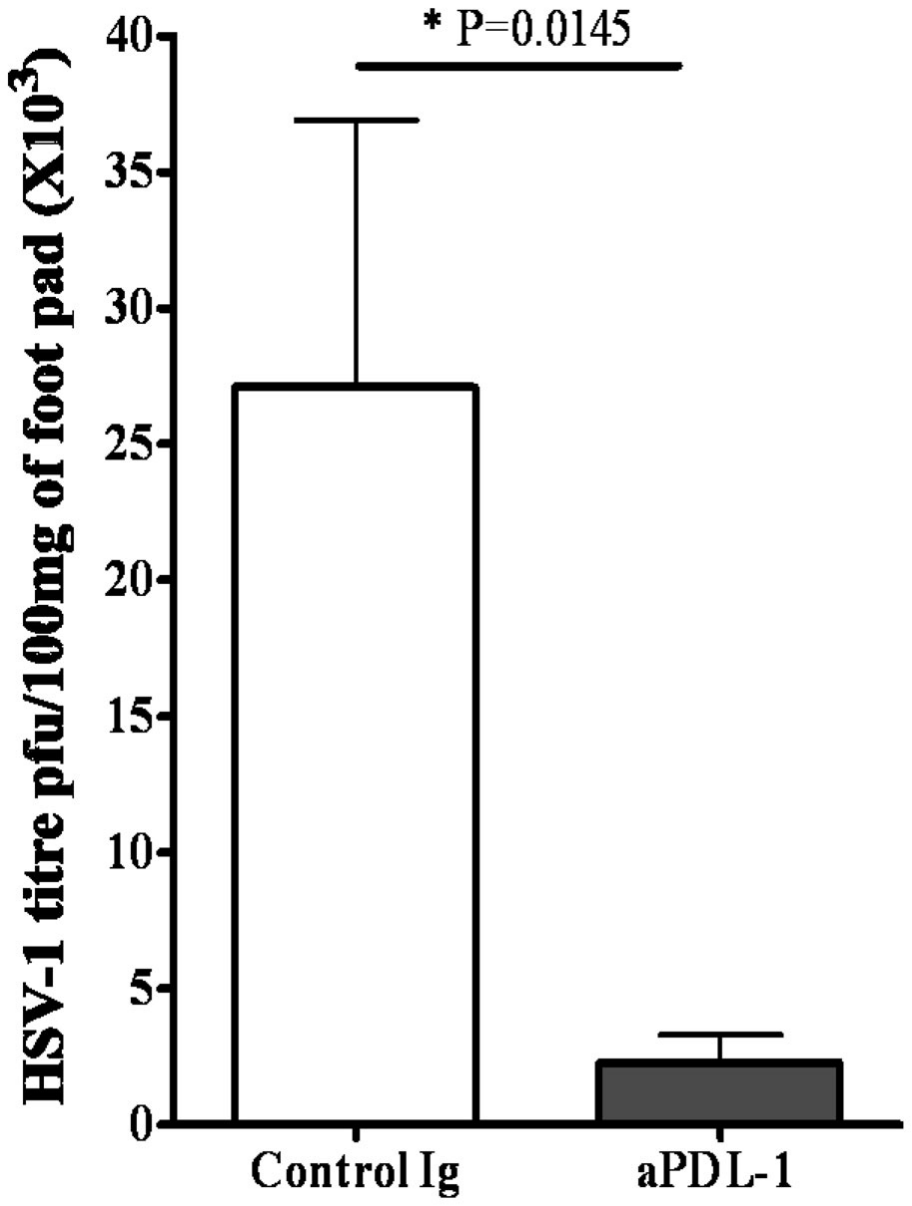
Enhanced viral clearance from the footpad of anti-PDL-1 treated group of mice when compared to isotype treated infected group. Mice were infected in the hind footpads with 1×10^6^ p.f.u of HSV-1. On day 6 post-infection, at the peak of the immune response, mice were euthanized. Skin tissue was removed from the footpad and weighed followed by homogenization in RPMI only. The supernatant obtained from the lysates was used in a Vero cell line based viral plaque assay to determine the virus titer. The results are expressed as number of plaque forming unit (p.f.u) of virus per 10 mg of footpad tissue. Error bar represents the mean ± S.D. of the viral plaques derived from the skin of six footpads per group. *p<0.05 compared with control HSV-infected B6 mice was considered statistically significant.

Our results showed that blocking the PDL-1 interaction at the time of priming increases both the number as well as the quality of SSIEFARL specific effector CD8 T cells in secondary lymphoid tissue (Figure 6 and 7). We next determined how enhanced CD8 T cell response affects the clearance of HSV-1 infection from the footpad of anti-PDL-1 treated group? Infected mice from the isotype and anti-PDL-1 treated groups were euthanized on day six post-infection and viral load in the foot-pad of infected mice was determined as described in the methods. As is shown in Figure 8, anti-PDL-1 antibody treatment resulted in a significantly lower level of HSV-1 in the footpad tissue when compared with the infected control group of mice at day 6 post-infection. These results highlight the effectiveness of systemic anti-PDL-1 antibody treatment in reducing the viral load during the acute phase of immune response to HSV-1.

### Blocking of PD-1: PDL-1 Interaction at the Time of Priming Enhances HSV-1 Specific Secondary CD8 T Cell Response in Spleen and Peripheral Blood

**Figure 9:**
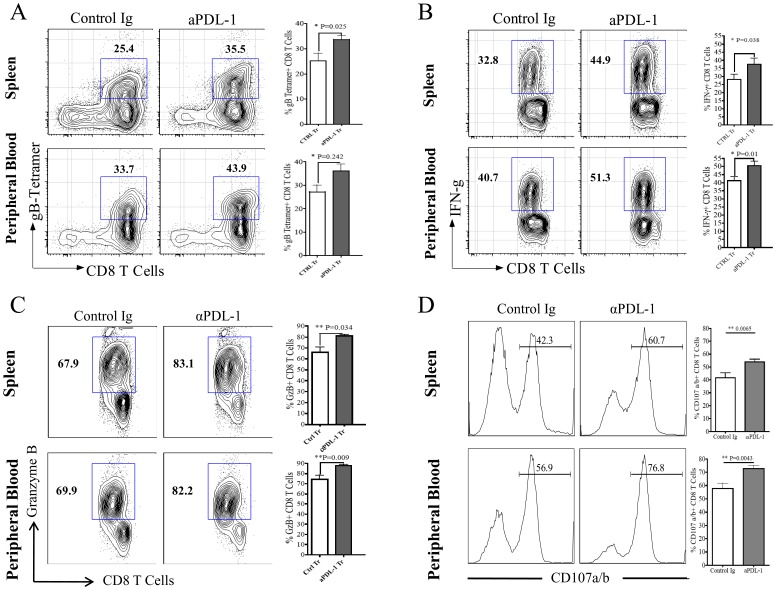
Anti-PDL-1 antibody treatment at the time of priming enhances secondary SSIEFARL specific CD8 T cell response. C57BL/6 mice were intra-peritoneally administered with either anti-PDL-1 or Isotype matched antibody on -1 and 3day post footpad HSV-1 infection. Thirty-two days post-infection both groups of mice were intra-peritoneally challenged with 1.5×10^6^ p.f.u of vaccinia virus encoding gB_498–505_ peptide. After 4 days of challenge, mice were euthanized and single cell suspension obtained from the spleen and peripheral blood of control and anti-PDL-1 antibody treated mice were used for flow-cytometeric studies. A, Representative FACS plots and bar graphs demonstrate the percentage of SSIEFARL Tetramer+ CD8 T cells in the spleen and peripheral blood of control and anti-PDL-1 treated groups of mice. B, FACS plots and bar graphs represent SSIEFARL specific IFN-γ producing memory CD8 T cells as determined by *ex-vivo* stimulation of the splenocyte and peripheral blood mononuclear cells (PBMC) obtained from control and anti-PDL-1 treated groups with gB_498–505_ peptide (10 µg/ml) for 6 h in presence of Brefeldin A. C, Representative FACS plots and bar graphs demonstrate the percentage of granzyme B expressing memory CD8 T cells in spleen and PBMC of control and anti-PDL-1 treated mice post-challenge. D, Histogram FACS plots, derived from gated CD8 T cell, and bar diagram represent the proportion of CD107a/b expressing memory CD8 T cells in spleen and peripheral blood of control and anti-PDL-1 treated groups of mice. Data shown is derived from two similar experiments with a total of five mice per group in each experiment. Statistical significance was determined by unpaired student’s t test where *p<0.05 and **p<0.01 are considered statistically significant.

The results shown above clearly demonstrate that anti-PDL-1 antibody treatment at the time of CD8 T cell priming effectively enhanced the HSV-1 specific effector CD8 T cell response in secondary lymphoid tissues. We next ascertained if PDL-1 blocking at the time of priming can also improve the quality of HSV-1 specific memory CD8 T cells. Anti-PDL-1 antibody was given one day before and three-day post footpad HSV-1 infection. The control groups of infected mice received isotype matched antibodies at the same time-points. On day 32 post-infection, both control and anti-PDL-1 antibody treated mice were intra-peritoneally challenged with 1.5×10^6^ p.f.u. of vaccinia virus encoding the SSIEFARL (gB_498–505_) peptide. On day four post-challenge, the proportion of SSIEFARL specific memory CD8 T cells and their quality was determined in the spleen and peripheral blood of both groups of mice. Upon recall, an increased proportion of SSEIFARL specific memory CD8 T cells were determined in the spleen and peripheral blood of the anti-PDL-1 treated group as compared to the isotype treated control group of infected mice (Figure 9A). In addition, the frequency of IFN-γ secreting memory CD8 T cells in response to *ex-vivo* stimulation with SSIEFARL peptide was significantly higher than in the anti-PDL-1 treated than the isotype treated group of infected mice (Figure 9B). Similarly, the frequency of CD8 T cells expressing granzyme B in the anti-PDL-1 treated group was significantly higher than in the isotype treated infected group (Figure 9C). Moreover, through the degranulation assay, we show that the memory CD8 T cells from the anti-PDL-1 treated group were more efficient in releasing granzyme B in response to *ex-vivo* stimulation with SSIEFARL peptide (Figure 9D). Thus, our results demonstrate that anti-PDL-1 antibody treatment at the time of priming increases the proportion of IFN-γ and granzyme B producing SSIEFARL specific memory CD8 T cells, and also increases the cytotoxic potential of HSV-1 specific memory CD8 T cell.

## Discussion

PD-1 is induced on CD8 T cells within 24–72 h of TCR stimulation. Earlier studies have suggested up-regulation of PD-1 on epitope-specific T cells after acute infection or DNA vaccination [13], [17]. Under chronic viral infection, PD-1 levels remain elevated on virus specific memory CD8 T cells, possibly in response to continuous antigenic stimulation. It has been reported that blocking of the PD-1 interaction with its ligand PDL-1 by using anti-PDL-1 antibody augments the memory CD8 T cell response to LCMV clone 13 and HIV infections [2], [4], [8]. However, how blocking the PD-1: PDL-1 interaction during the acute phase of immune response affects the quality of virus specific effector or memory CD8 T cell is somewhat controversial and not clear. In this report, using cutaneous HSV-1 infection model, we showed that PD-1 is upregulated on gB_498–505_ tetramer specific CD8 T cells after footpad HSV-1 infection in the draining popliteal lymph node and spleen tissue. A small proportion of antigen specific CD8 T cells expressed higher levels of PD-1 whereas the rest expressed medium levels of PD-1. In a similar study using the Friend Retrovirus infection model, a differential level of PD-1 expression on virus specific CD8 T cells during the acute phase of immune response was observed [13]. In that particular study, PD-1^high^ CD8 T cells produced less pro-inflammatory cytokines but had more cytotoxic potential than PD-1^low^ cells upon *ex-vivo* antigenic stimulation. In our infection model, PD-1^high^ CD8 T cells produced less pro-inflammatory cytokines IL-2, IFN-γ and TNF-α in response to *ex-vivo* stimulation with immunodominant SSIEFARL peptide as compared to the PD-1^medium^ CD8 T cell pool. In addition, PD-1^high^ CD8 T cells were less cytotoxic in comparison to PD-1^medium^ CD8 T cells in the spleen as determined by the CD107 degranulation assay. Our results suggest that PD-1^high^ CD8 T cells during the lytic phase of HSV-1 infection were more exhaustive than PD-1^medium^ CD8 T cells. A recent study has also demonstrated the inhibitory effect of PD-1 on CD8 T cells during the lysogenic phase of HSV-1 infection in the trigeminal ganglia [18]. In chronic LCMV infection, PD-1 was differential expressed on PD-1 were noticed on virus specific memory CD8 T cells, and PD-1^high^ cells were considered terminally differentiated as they respond poorly to the blocking of PD-1: PDL-1 interaction [19].

To regulate CD8 T cell effector function, PD-1 expressed on CD8 T cells must interact with its respective ligand PDL-1 expressed on hematopoietic cells in the presence of antigenic stimulation [15]. Dendritic cells are the key players in priming HSV-1 specific CD8 T cells in the draining lymph node [16]. We determined that PDL-1 is transiently upregulated on DCs after HSV-1 infection. Following T cell activation, many co-stimulatory and inhibitory receptors are upregulated on T cells and it is possible that inhibitory interactions between PD-1 on T cells and PDL-1 on DCs at the time of priming are attenuating the effector T cell response. In fact, Mueller *et al* have shown that LCMV clone 13 infection of mice lacking PDL-1 expression on hematopoietic cell types generates more IFN-γ and TNF-α secreting virus specific CD8 T cells on day 8 post-infection as compared to PDL-1 expressing infected mice [15]. In a study of rabies virus infection in mice, the PD-1: PDL-1 interaction was shown to dampen the virus specific effector CD8 T cell response in order to protect the central nervous system (CNS) from immunopathology [20]. Similarly, in response to influenza virus infection, PDL-1−/− mice develop fewer virus specific CD8 T cells as compared to the infected wild type [21]. Our results are in agreement with these studies as we determined that blocking the PDL-1 interaction *in vivo* by administering anti-PDL-1 antibody at the time of T cell priming enhances the number of SSIEFARL specific CD8 T cells in secondary lymphoid tissues. We also determined that blocking the PD-1: PDL-1 interaction at the time of priming increases the frequency of cytokine secreting SSIEFARL specific CD8 T cells and improves their cytotoxic potential. In contrast, in an acute FIV infection model, PDL-1 blocking did not significantly improve effector CD8 T cell response [13]. This discrepancy could be due to little or less upregulation of inhibitory PDL-1 on hematopoietic cells after FIV infection. Moreover, the lesser amounts of PDL-1 may not effectively exert its inhibitory effect in the presence of strong co-stimulation in FIV infection model.

The PD-1: PDL-1 interaction has also been shown to regulate the effector function of HSV-1 specific CD4 T cells in an ocular HSV-1 infection study [22]. In that particular study, *in vivo* blocking of the PD-1: PDL-1 interaction enhanced the pathogenesis of herpetic stromal keratitis (HSK). However, a more recent study showed that in response to a series of *in vivo* administered anti-PDL-1 antibody, given after ocular HSV-1 infection, the proportion of SSIEFARL specific CD8 T cells decreased in the draining lymph node when measured at day 7 post-infection [23]. In addition, anti-PDL-1 antibody treatment also resulted in higher viral load in the cornea and TG at day 7 post-infection. Our results are in contrast to this particular study as we determined that anti-PDL-1 antibody treatment significantly increased the numbers and effector function of SSIEFARL specific CD8 T cells. The major difference between our study and the study mentioned above is the route of infection (footpad versus ocular) and the course of anti-PDL-1 antibody treatment. In our study, anti-PDL-1 antibody was given one day prior to infection followed by a second treatment on day three post-infection, whereas in the study mentioned above, injections were administered on days 2, 4 and 6 post-infection. Since, the peak of PDL-1 expression occurred on day 2 post-infection (Figure 4), it is possible that early or late blocking of the PD-1: PDL-1 interaction might have a differential outcome on the CD8 T cell response to HSV-1 infection.

CD8 T cells are well known to play an important role in regulating HSV-1 latency in the trigeminal ganglia (TG) [24]. Several recent studies have characterized the role of PD-1 in causing the functional exhaustion of HSV specific CD8 T cells in the TG [18], [25], [26], [27]. One such study demonstrates that HSV-1 latency associated transcript (LAT) promotes the functional exhaustion of virus specific CD8 T cells by inducing PD-1 expression [26]. The study demonstrates that mice infected with LAT^(+)^ HSV-1 harbor more PD-1 expressing HSV specific CD8 T cells as compared to mice infected with LAT^(−)^HSV-1. Moreover, PD-1 expressing CD8 T cells produced less cytokines in response to stimulation with SSIEFARL peptide suggesting the involvement of PD-1 in regulating effector function of CD8 T cells. In fact, the absence of PD-1 results in a significantly reduced amount of HSV-1 latency [18]. Our results are in agreement with these studies demonstrating the ability of PD-1 to regulate effector function of HSV-1 specific CD8 T cells.

It is well established that programming to develop good quality virus specific memory CD8 T cells occur during first few days of infection [28]. A recent study has shown that memory CD8 T cells generated in PD-1−/− mice, upon recall, respond better in comparison to cells generated in wild type control mice, suggesting that PD-1 signaling is important for regulating the quality of the memory CD8 T cell pool [29]. However, in this study, PD-1 signaling was absent from the beginning to the end of the experiments. We blocked the PD-1: PDL-1 interaction only at the time of priming, the time-point when the programming of memory CD8 T cells occurs. This resulted into the generation of memory CD8 T cells that upon recall with vaccinia virus encoding gB_498–505_, produced more IFN-γ and granzyme B in comparison to memory CD8 T cells that were developed in the presence of PD-1: PDL-1 interaction. Even though PDL-1 blocking at the time of priming significantly improved the quality of HSV-1 specific memory CD8 T cells, the increase noted in our study was not dramatic. One possible explanation is that other inhibitory receptors like CTLA-4 and Tim-3 that are also upregulated on CD8 T cells (data not shown) might be exerting their effects at the time of priming. In fact, both CTLA-4 and Tim-3 have been shown to work collectively with PD-1 to control memory CD8 T cell function during chronic hepatitis C virus and LCMV infections [30], [31]. It is also possible that certain other inhibitory receptors like CD160, LAG3, BTLA and IL-10R might be involved in regulating the effector function of HSV specific CD8 T cells.

Taken together, this study supports the notion that blocking the PDL-1 interaction at the time of priming enhances the primary and secondary CD8 T cell responses to acute virus infection. This suggests that targeting the PD-1: PDL-1 interaction in combination with other approaches could be used to enhance the epitope specific T cell response in a prophylactic vaccination system. In fact, a study carried out in rhesus macaques has shown that blocking the PD-1: PDL-1 interaction at the time of vaccination with an adenovirus vector encoding SIV-gag effectively increases the number of gag-specific T cells [32]. However, caution should be taken as an over-active effector CD8 T cell response in the complete absence of the PD-1: PDL-1 interaction might also cause tissue damage as reported in chronic LCMV clone 13 infection [15].

## Materials and Methods

### Ethical Statement

All the experiments carried out in this manuscript were in accordance with the approval obtained from the members of the Institutional Animal Care Committee (IACUC) of Oakland University (Approval number 10102). Mice were housed in the Biomedical Research Support Facility (BRSF) at Oakland University as per Association for Assessment and Accreditation of Laboratory Animal Care International (AAALAC) approved guidelines.

### Mice, Virus and Reagents

Female C57BL/6J mice (8–12 weeks) were purchased from The Jackson Laboratories. The HSV-1 (KOS) strain was obtained from Dr. Robert Hendricks at The University of Pittsburgh School of Medicine, Pittsburgh, PA. The virus was propagated on monolayer of Vero cells (CCL81) as described earlier. Vero cells were obtained from American Type culture collection (ATCC), Manassas, VA. HSV-1 was titrated and stored in aliquots at −80°C until further use. Recombinant vaccinia virus encoding gB_498–505,_ provided by Dr. S.S. Tevethia, was used to measure the recall response of SSIEFARL specific memory CD8 T cells. The gB_498–505_ (SSIEFARL) peptide was synthesized and supplied by Research Genetics, Huntsville, AL. APC-anti-CD44, Percp-cy5.5 or PE-anti-IFN-γ, APC-anti-TNF-α, APC-anti-IL-2, FITC-anti-CD107a/b, and Percp-cy5.5-anti-CD11c antibodies were procured from BD biosciences. PE-cy7-anti-CD8, FITC or PE-anti-PD-1 and PE-anti-PDL-1 antibodies were purchased from e-biosciences.

### HSV-1 Infection and anti-PDL-1 Antibody Treatment

C57BL/6J mice were infected subcutaneously in the footpad of the hind legs with 1×10^6^ plaque forming units (p.f.u) of HSV-1 (KOS). Infected mice received, intra-peritoneally, 300 µg of anti-PDL-1 (10F.9G2) or Rat IgG monoclonal antibody (mAb) per mouse on −1 and +3 days post infection. On day 6 post-infection, mice were euthanized and the HSV-1 specific CD8 T cell response was measured by different *ex-vivo* immunological assays in the PLN and spleen tissue of anti-PDL-1 and isotype treated groups of infected mice. All the experiments were carried out minimally two times or as stated in the figure legends.

### Tetramer Staining and Flow Cytometery

MHC class I (H-2^b^) tetramers to measure SSIEFARL specific CD8 T cells were obtained from the NIH tetramer core facility at Atlanta, GA. A total of 10^6^ cells obtained from PLN and spleen of HSV-1 infected mice were first incubated on ice with Fc block (BD Biosciences) for 25 minutes followed by incubation with PE-conjugated SSIEFARL tetramer at 37°C for 30 min. Cells were then surface stained for CD8 and PD-1 molecules in FACS buffer on ice. FACS Canto II (BD Biosciences) was used to acquire the samples and the data was analyzed by Flowjo software (Treestar Inc., Ashland, OR).

### PDL-1 Staining on CD11C+ Dendritic Cells in Popliteal Lymph Node

Popliteal lymph nodes were obtained on different days post-infection from C57BL/6J mice. PLNs were digested with type IV collagenase (400 u/ml, Sigma) and DNase I (50 µg/ml, Roche) for 25 minutes at 37°C. Single cell suspension obtained were surface stained for CD11c and PDL-1 while using Fluorochrome conjugated anti-CD11c and PDL-1 antibodies (BD biosciences and e-biosciences respectively). Samples were acquired on a FACS Canto II and analyzed with Flowjo software.

### Intracellular Cytokine Staining (ICS) Assay

To determine the cytokine expression profile of PD-1 expressing CD8 T cells in anti-PDL-1 treated and isotype treated groups of infected mice, 1×10^6^ cells obtained from PLN and spleen tissue were cultured in the presence or absence of SSIEFARL peptide (10 µg/ml) for 5 h with Brefeldin A (10 µg/ml) added during last 3 h of incubation at 37°C. Cell surface staining for CD8 and PD-1 was carried out followed by intra-cellular cytokine staining for IFN-γ, TNF-α and IL-2 by using Cytofix/Cytoperm kit (BD Biosciences) as per the manufacturer’s instructions.

### Measurement of Cytotoxic Potential of SSIEFARL Specific CD8 T Cells

The cytotoxic potential of HSV-1 specific CD8 T cells in ant-PDL-1 and isotype antibody treated groups of infected mice was determined by a degranulation assay as described previously [33], [34]. Briefly, single cell suspensions of spleen and PLN were stimulated with SSIEFARL peptide for 5 h in the presence of Fluorochrome labeled anti-CD107a/b antibodies. Brefeldin A was added during the last 3 h of incubation at 37°C. At the end of the incubation period, cell surface staining for CD8 was carried out, followed by intra-cellular staining (using Cytofix/Cytoperm kit) for granzyme B molecule. The cytotoxic potential of PD-1^high^ and PD-1^medium^ CD8 T cells after HSV-1 infection was also determined by the degranulation assay described.

### Quantitation of HSV-1 in Footpad Tissue

The quantitation of HSV-1 in footpad tissue was determined as previously reported [35]. Briefly, anti-PDL-1 and isotype antibody treated groups of infected mice were euthanized at day 6 post-infection. Skin tissues were removed from the hind footpad of euthanized mice and a total of 100 mg of tissue was collected in 1 ml of RPMI only medium. Homogenization of the collected tissue was done in 1 ml glass homogenizers (Wheaton) on ice. The lysates were centrifuged at 4°C and the supernatant was used to assay the amounts of virus on Vero cell line. Plaques were determined by counter-staining the cell monolayer with Coomassie blue R-250 (GibcoBRL) as described earlier [35].

### Recall Response of HSV-1 Specific Memory CD8 T Cells

To determine the quality of HSV-1 specific memory CD8 T cell generated in anti-PDL-1 and isotype treated groups, infected mice were challenged intra-peritoneally with 1.5×10^6^ p.f.u of vaccinia virus encoding SSIEFARL peptide (VV_gB498–505_) on day 32 post-infection. On day 4 post-challenge, mice were euthanized, and the quantity and quality of SSIEFARL specific memory CD8 T cells was determined in the spleen and peripheral blood of both groups of mice. Each group was comprised of four to five C57BL/6 mice.

### Statistical Analysis

Data was analyzed using Student’s t test in Graph pad Prism Software. p values of *p≤0.05, **p≤0.01 and ***p≤0.001 were considered statistically significant. Results in the graphs are represented as mean ± S.D. unless otherwise mentioned.
